# LncRNA MALAT1 facilitates lung metastasis of osteosarcomas through miR-202 sponging

**DOI:** 10.1038/s41598-020-69574-y

**Published:** 2020-07-29

**Authors:** Jun Zhang, Cheng-Dong Piao, Jie Ding, Zheng-Wei Li

**Affiliations:** 1 Department of Orthopedics, The Second Hospital of Jilin University, 218 Ziqiang Street, Nanguan District, Changchun, 130041 Jilin People’s Republic of China; 2 Department of Stomatology, The Affiliated Hospital of Changchun University of Traditional Chinese Medicine, 1478 Gongnong Street, Chaoyang District, Changchun, 130021 Jilin People’s Republic of China

**Keywords:** Bone cancer, Bone cancer

## Abstract

Lungs are the primary metastatic sites for osteosarcomas responsible for associated mortality. Recent data has documented role of long non-coding RNAs (lncRNAs) in proliferation and growth of osteosarcoma cells. We evaluated a role of lncRNAs in the lung metastasis of osteosarcoma with the goal of identifying a unique signature. Comparison of different lncRNAs in tumor samples from osteosarcoma with and without lung metastasis led to identification of MALAT1 as the most differentially upregulated lncRNA in the osteosarcoma patients with lung metastasis. MALAT1 was also high in osteosarcoma cells KRIB and MALAT1’s targeted downregulation in these cells led to decreased invasive potential and identification of miR-202 as the miRNA that is sponged by MALAT1. In the lung metastasis in vivo model, parental KRIB cells metastasized to lungs and such metastasis was significantly inhibited in KRIB cells with downregulated MALAT1. Ectopic miR-202 expression attenuated KRIB downregulation-mediated effects on lung metastasis. In yet another in vivo model involving parental SAOS-2 and lung-metastatic derivatives SAOS-2-LM, MALAT1 expression was found to be elevated in lung metastatic cells, which also correlated with reduced miR-202. In conclusion, MALAT1-miR-202 represents a potential lncRNA-miRNA signature that affects lung metastasis of osteosarcomas and could potentially be targeted for therapy.

## Introduction

Osteosarcoma, also referred to as osteogenic sarcoma, is a malignant bone tumor primarily found in extremities, but may also be present in the axial skeleton. In comparison to other more prevalent cancers such as lung cancer, breast cancer etc., it is a rather ‘rare’ cancer with a worldwide incidence of 3.4 cases per million per year^1–3^. Even though osteosarcoma is rare at ages below five^4^, its incidence in children between the ages of 5 and 15 is 5.6 cases per million per year^2^. Thus, osteosarcoma is more prevalent at adolescence. In the United States of America, the estimated incidence of osteosarcoma is 800–900 new cases per year with half of these diagnoses in children and teens^1^. In China as well, osteosarcoma is similarly rare^5^, trending almost similarly as the rest of the world. Although known for diagnoses at early ages, osteosarcoma can really be diagnosed at any age, even at substantially older ages. Extraskeletal osteosarcoma, similarly rare but, in contrast with the classical osteosarcoma, is often diagnosed at a median age of 55.5 years of age in China, and is highly metastatic^6^.

Treatment of osteosarcoma requires a multidisciplinary approach involving multiple specialists such as a primary care physician, an orthopedic oncologist, a medical oncologist, a radiologist and a pathologist^7^. Such collaborative efforts have led to substantial gains, with 5-year survivals for osteosarcoma reported to be between 70 and 80%^8^. However, there is need to further improve the patient outcomes. Osteosarcomas commonly metastasize to lungs^9–11^ and such lung metastases of osteosarcomas present one area of focus for future studies. Of note, the 5-year survival for patients with metastatic disease is much reduced and only around 10–30%^12^.

Based on the available literature, it is obvious that treatment of lung metastasis of osteosarcoma can substantially increase the prognosis. Towards this goal, it is prudent to first understand the mechanism of lung metastasis of osteosarcoma. In this work, we focused on lncRNAs as the factors that could play a role in lung metastasis of osteosarcoma. In recent years, there has been an interest in the role of lncRNAs in the progress of osteosarcoma thus raising the possibility of them being used as therapeutic targets^13^. We screened a number of lncRNAs in the patient samples representing osteosarcoma patients without and with lung metastasis. We also provide a mechanism of lncRNA functioning through miRNA sponging.

## Materials and methods

### Osteosarcoma patients

The patients reported to our Jilin University Hospital and were enrolled with informed consent starting in July 2017 and ending in January 2019. The Ethics Committee at the Jilin University (Approval Number 17/1089) reviewed and approved the study. Inclusion criteria was positive and primary diagnosis of osteosarcoma. In the patients with metastasis, inclusion criteria was the diagnosis of only lung metastasis. The research team had no information on the identity of research participants. 32 osteosarcoma without metastasis and 24 osteosarcoma patients with lung metastases were made part of the study as they met the inclusion criteria. In addition, archived tissues from 30 control individuals with no osteosarcoma diagnosis were used for diagnostic utility of lncRNA. All samples were stored in a − 80 °C freezer. The gender and age distribution of patients is provided in Table 1.

**Table 1:** Gender and age details of patient.

Group	N	Age range (years)	Mean age (years)	Males (%)	Females (%)
Osteosarcoma (no metastasis)	32	15–40	22	20 (62.50)	12 (37.50)
Lung metastatic osteosarcoma	24	20–45	29	16 (66.67)	8 (33.33)
Controls	30	15–45	25	20 (66.67)	10 (33.33)

### Cell lines

Osteosarcoma cell lines KRIB, SaOS, MG63 and U2OS were purchased from American Type Culture Collection (ATCC, USA). Cells were cultured in DMEM medium supplemented with 10% fetal bovine serum at 37 °C. hFOB 1.19 cells were also purchased from ATCC (USA) and cultured in F12-DMEM 10% fetal bovine serum and 0.3 mg/ml G418. All cells were grown in a controlled atmosphere containing 5% CO_2_.

### RNA preparation

RNAiso reagent (TaKaRA, China) was used to isolate RNA, as per the manufacturers’ instructions. Quality of RNA was checked on a Nanodrop 2000 instrument (ThermoFisher Scientific, China). Samples with OD_260_/OD_280_ > 1.8 were used for further analysis. Integrity of RNA was also checked using Bioanalyzer (Agilent Technologies, Japan) that utilized an RNA 6,000 Nano LabChip.

### Quantitative RT-PCR

All primers and detection reagents for the detection of lncRNAs were from Qiagen (China). RNAse-free water was used for the experiments. RT^2^ first strand kit (Qiagen, China) was used for the synthesis of cDNA. Quantity of RNA was 1 μg and 2 μl of genomic DNA elimination mix was added and mixed, followed by incubation for 5 min at 42 °C and then quick transfer to ice-cold water for 1 min. Reverse transcription mix, consisting of 5 × buffer and reverse transcriptase enzyme, was then added and incubated for 15 min at 42 °C. At the end of incubation, reaction was stopped by placing the reaction mixture containing tube at 95 °C. All lncRNAs were detected using probes from Qiagen (China). qPCR for miRNAs was conducted using probes and primers from Thermo Scientific Fisher (China) according to manufacturer’s instructions. Results were normalized using glyceraldehyde-3-phosphate dehydrogenase (GAPDH) or U6 as an internal control.

### MALAT1 downregulation

MALAT1 downregulation was achieved using locked nucleic acid GapmeR from Qiagen (China) with the sequence:5′-AGATTCCGTAACTTTA-3′. For control conditions, a control LNA GapmeR was used with the sequence: 5′-AACACGTCTATACGC-3′. KRIB cells were transfected at ~ 50% confluency with 20 nM LNA GapmeRs, using Lipofectamine RNAiMax (Thermo Fisher Scientific, China).

### microRNA mimics and inhibitor transfections

miRNA mimics (GenePharma, China) with corresponding control RNA were transfected, using Lipofectamine 2000 transfection reagent (Thermo Fisher Scientific, China), in osteosarcoma cells when the cells were in logarithmic growth phase. The protocol suggested by the manufacturer was followed. For the inhibition of miRNAs, specific inhibitors or non-specific controls were purchased from Thermo Fisher Scientific (China) which were transfected in cells, using Lipofectamine reagent (Thermo Fisher Scientific, China).

### Invasion assay

For evaluation of invasion potential, cells were trypsinized, collected, resuspended in serum-free medium and counted. 1 × 10^5^ cells were seeded into a Matrigel (BD Bioscience, China) chamber (Corning, China), and the chamber was placed on a well containing normal culture media with 10% serum. After 20 h of growth. Cells still in the Matrigel were removed using a cotton swab, and the cells that had invaded through the Matrigel and were now on the lower membrane surface were fixed with 4% paraformaldehyde and stained with 0.1% crystal violet. Cells were then counted under a bright field microscope by two independent personnel.

### In vivo lung metastases

The animal experiments described in the manuscript were approved by the Animal Research Ethics Committee at the Jilin University. All methods were performed in accordance with the relevant guidelines and regulations. The mice were housed in sterilized animal facility and food and water was provided ad libitum. For in vivo lung metastasis, 10^6^ KRIB cells were injected into the tail veins of BALB/c nude male mice (Shanghai SLAC Laboratory Animals Co., Ltd., Shanghai, China) by i.v. injections. After 6 weeks, pulmonary metastatic lesions were manually counted independently by two laboratory personnel and cross-verified.

### Nude mouse model of human osteosarcoma lung metastases

We developed a nude mouse model for the study of human osteosarcoma metastases, based on the study by Jia et al.^14^. Briefly, 10^6^ SAOS-2 cells were i.v. injected into the tail veins of BALB/c nude male mice (Shanghai SLAC Laboratory Animals Co., Ltd., Shanghai, China). Pulmonary metastatic lesions were surgically removed and cultured in laboratory as single cell suspensions using the cell culture conditions for SAOS-2 cells for 2 passages, after which they were re-injected into mice i.v. 10^6^ per mice, and the metastatic lesions again surgically removed and cultured in the laboratory. After 3 such cycles, cells isolated from pulmonary metastases were used for molecular analysis.

### Statistical considerations

Date was analyzed by two biostatisticians. The identity of samples was not revealed to the biostatisticians. *p* value was calculated for the comparison of 2 independent groups using Student’s *t* test, one-way ANOVA, and Pearson’s correlation analysis. Representative results from three repeats are presented. Only the *p* values ≤ 0.05 were considered to represent statistically significant analyses.

## Results

### Dysregulated lncRNAs in lung metastatic osteosarcomas

**Figure 1:**
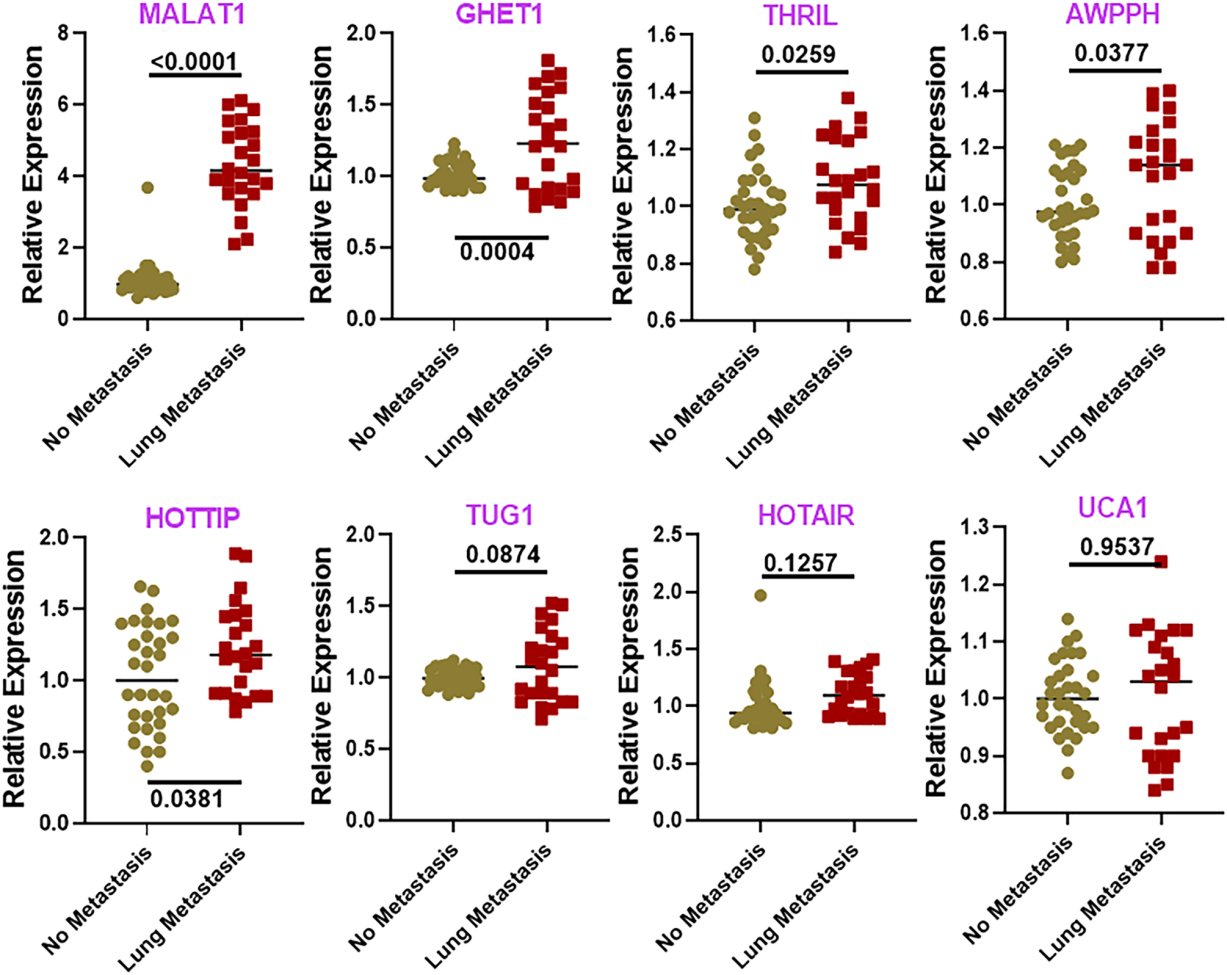
Upregulated lncRNAs in lung metastatic osteosarcoma. Levels of lncRNAs were evaluated in tumor samples collected from patients without lung metastasis (n = 32) versus patients with lung metastasis (n = 24), using qRT-PCR. lncRNA levels in the No metastasis group were collectively set to have a mean of 1.0 and relative levels in lung metastasis group are plotted. *p* values determining the level of statistical significance are mentioned for all groups. No metastasis: Osteosarcoma patients with no metastasis. Lung metastasis: Osteosarcoma patients with lung metastasis.

**Figure 2:**
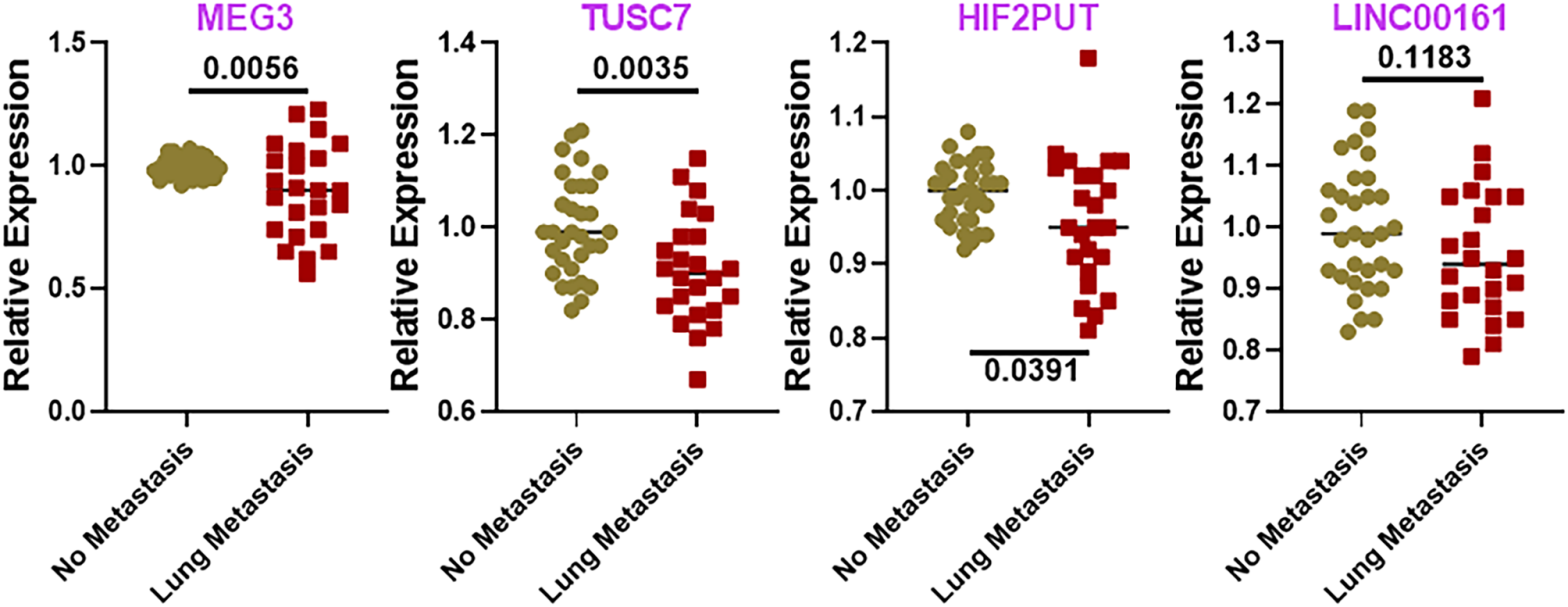
Downregulated lncRNAs in lung metastatic osteosarcoma. Levels of lncRNAs were evaluated in tumor samples collected from patients without lung metastasis (n = 32) versus patients with lung metastasis (n = 24), using qRT-PCR. lncRNA levels in the No metastasis group were collectively set to have a mean of 1.0 and relative levels in lung metastasis group are plotted. *p* values determining the level of statistical significance are mentioned for all groups. No metastasis: Osteosarcoma patients with no metastasis. Lung metastasis: Osteosarcoma patients with lung metastasis.

With the objective of finding an lncRNA signature for osteosarcomas that metastasize to lungs, we evaluated the expression levels of several lncRNAs in patient samples. We compared lncRNAs in samples of patients with lung metastases versus samples of patients with no metastasis. A number of lncRNAs were shortlisted for evaluation based on the published reports. Some lncRNAs have been evaluated earlier in osteosarcoma models while the other tested lncRNAs have been reported in other cancer models. Several lncRNAs were significantly upregulated (Fig. 1) as well as downregulated (Fig. 2) in lung metastatic osteosarcomas.

MALAT1 was revealed to be the most significantly upregulated lncRNA with a *p* value of < 0.0001. A number of other lncRNAs were also significantly upregulated—GHET1, THRIL, AWPPH and HOTTIP (Fig. 1). In addition, we found that some other lncRNAs were also upregulated that did not reach statistical significance. These included lncRNAs TUG1, HOTAIR and UCA1 (Fig. 1) which were slightly elevated in lung metastatic osteosarcomas. Among the downregulated lncRNAs, MEG3 was determined to be the most downregulated with a *p* value of 0.0056. TUSC7 and HIF2PUT lncRNAs were also significantly downregulated (Fig. 2). Additionally, LINC00161 was found to be downregulated but could not reach statistical significance (Fig. 2).

### In-vitro osteosarcoma model for lncRNA study

**Figure 3:**
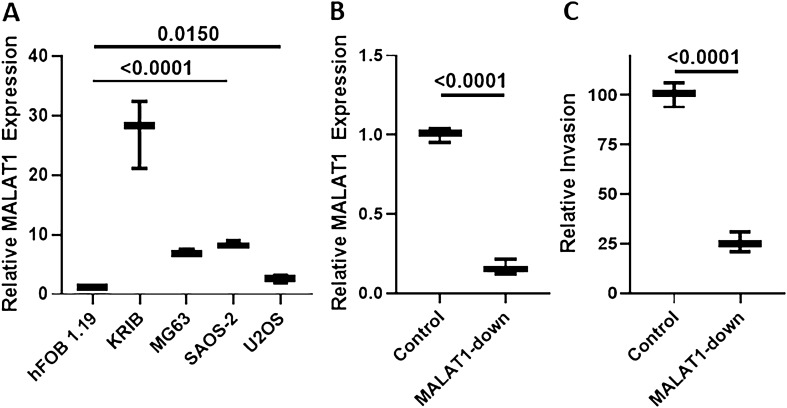
In vitro effects of MALAT1 downregulation. (**A**) Levels of MALAT1 were evaluated by qRT-PCR in osteosarcoma cells KRIB, MG63, SAOS-2 and U2OS, and compared to the levels in hFOB 1.19. MALAT1 levels in hFOB 1.19 cells were set to have a mean of 1.0 and relative levels in osteosarcoma cells are reported. (**B**) MALAT1 was downregulated in KRIB cells and the efficiency of downregulation confirmed by qRT-PCR. MALAT1 levels in control cells were set to have a mean of 1.0 and relative levels in KRIB cells with downregulated MALAT1 are reported. (**C**) Invasive potential of KRIB cells, control and MALAT1 downregulated, was also evaluated. Invasion by control cells was set to have a mean of 100 and relative invasion by KRIB cells with downregulated MALAT1 is reported. MALAT1-down: KRIB cells with downregulated MALAT1.

For performing experiments to study mechanism, we evaluated MALAT1 levels in several osteosarcoma cell lines so as to find the most appropriate model. Through this screening, we found that KRIB cells had the highest endogenous levels of MALAT1, compared to the hFOB 1.19 control human osteoblast cells (Fig. 3A). A few other commonly studied osteosarcoma cells also had higher MALAT1 levels than hFOB 1.19 cells, such as, MG63, SAOS-2 and U2OS but the MALAT1 levels were clearly the highest in KRIB cells.

To understand if MALAT1 can indeed effect cancer cell metastasis, we performed invasion assay using KRIB cells. First, we downregulated MALAT1 in KRIB cells and checked for the efficiency of downregulation. MALAT1 was very efficiently and significantly downregulated in KRIB cells (Fig. 3B), therefore, we used this model for evaluation of invasive potential. While the control KRIB cells with normal MALAT1 levels were highly invasive, those with downregulated MALAT1 were substantially less invasive (Fig. 3C).

### microRNA sponging by MALAT1

**Figure 4:**
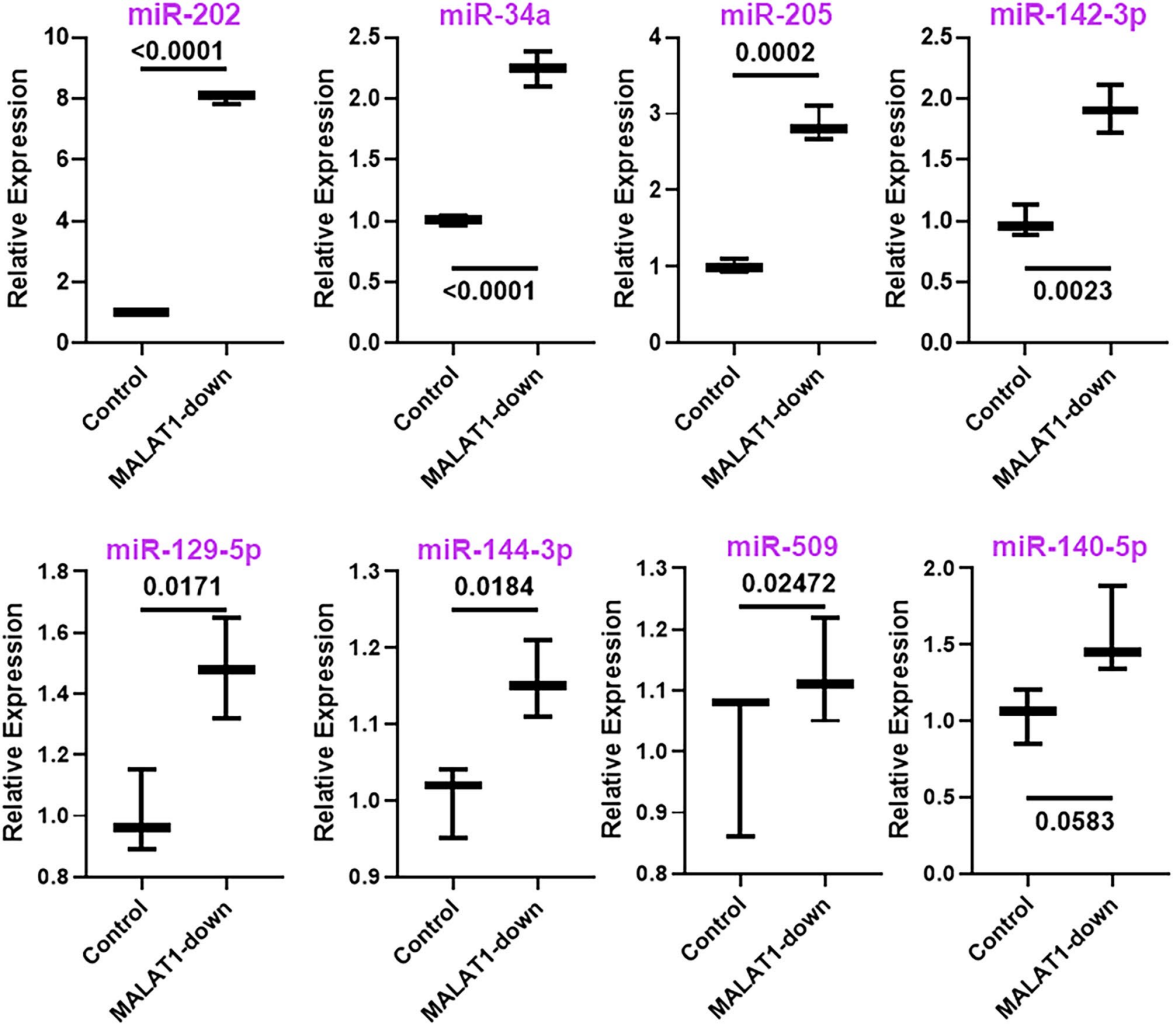
miRNAs regulated by MALAT1. Levels of several MALAT1 regulated miRNAs were evaluated in KRIB cells after MALAT1 down-regulation, using qRT-PCR. miRNA levels in the control KRIB cells were set to have a mean of 1.0 and relative levels in MALAT1 downregulated cells are plotted. *p* values determining the level of statistical significance are mentioned for all groups. MALAT1-down: KRIB cells with downregulated MALAT1.

LncRNAs function via sponging their target microRNAs (miRNAs)^15^. Therefore, we were interested in finding a miRNA that was functionally involved in lncRNA MALAT1’s actions towards promoting lung metastasis of osteosarcoma. We turned to both published reports as well as online tools to predict miRNA targets of MALAT1. Many such potential miRNAs were evaluated as presented in Fig. 4. Two miRNAs, miR-202 and miR-34a stood out as the most significantly (*p* < 0.0001) upregulated miRNAs in KRIB cells that had MALAT1 downregulated. In addition, several other miRNAs, such as, miR-205, miR-142, miR-129, miR-144 and miR-509 were also significantly upregulated but the levels of miR-202 were found to be the highest—~ eightfolds higher than the control cells. miR-140-5p was also elevated but did not reach statistical significance (Fig. 4).

### In vivo findings

**Figure 5:**
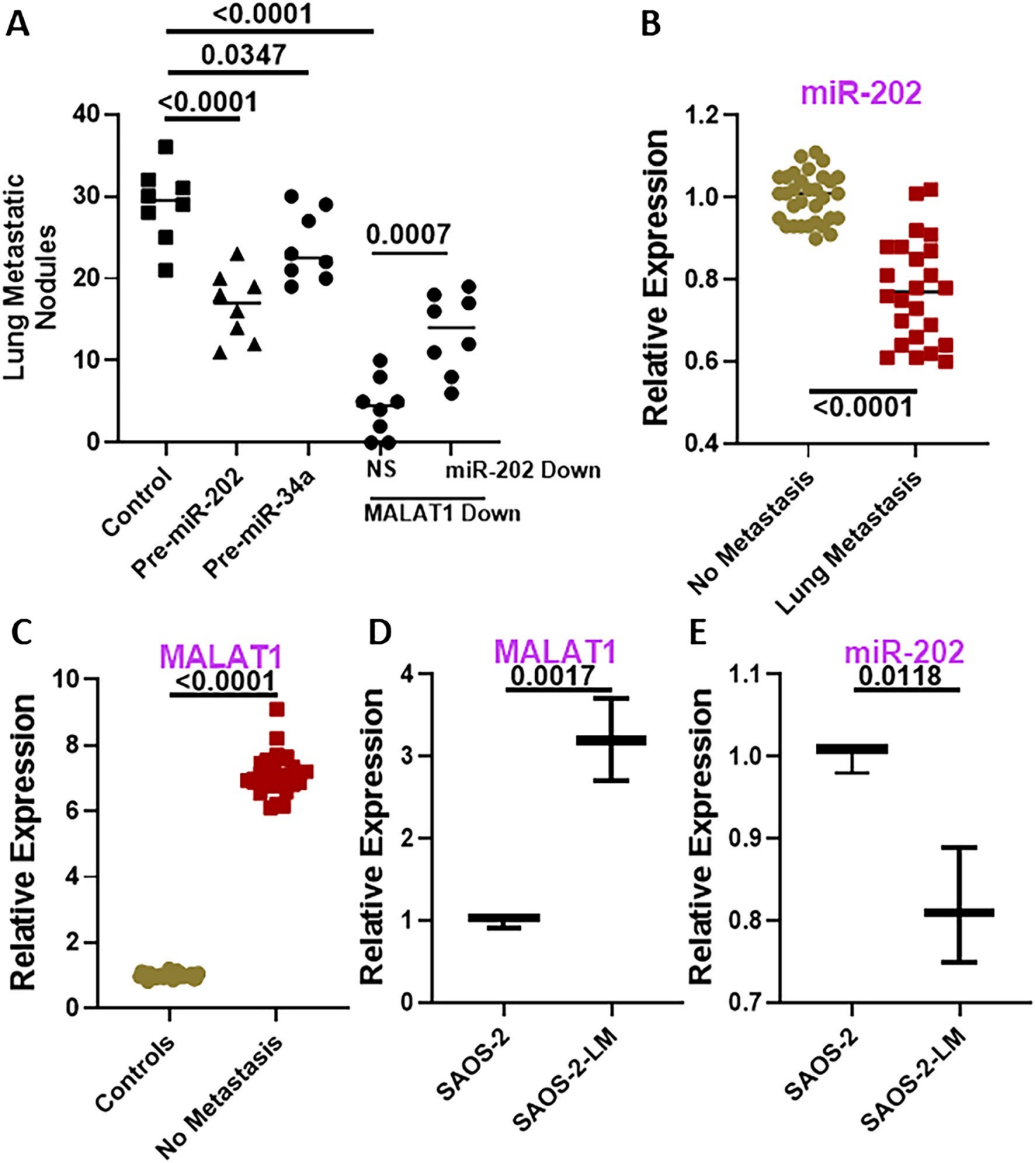
Lung metastasis models. (**A**) Lung metastatic modules were counted after excision of lungs of animals that were injected i.v. with KRIB cells. Effect of MALAT1 downregulation and the attenuating effect of miR-202 on metastatic nodules was evaluated by injection of KRB cells with downregulated MALAT1 without or with pre-miR-202. n = 8 mice per group. (**B**) Levels of miR-202 were evaluated in tumor samples collected from patients without lung metastasis (n = 32) versus patients with lung metastasis (n = 24), using qRT-PCR. miR-202 levels in the No metastasis group were collectively set to have a mean of 1.0 and relative levels in lung metastasis group are plotted. (**C**) Levels of lncRNA MALAT1 were evaluated in tumor samples collected from patients without lung metastasis (n = 32) versus archived tissues samples from control healthy individuals with no osteosarcoma diagnosis (n = 30), using qRT-PCR. MALAT1 levels in the control healthy group were collectively set to have a mean of 1.0 and relative levels in patients group are plotted. We also created an SAOS-2 cells’ lung metastatic model as described in Methods and evaluated (**D**) MALAT1 and (**E**) miR-202 levels in parental SAOS-2 and lung metastatic SAOS-2 cells (SAOS-2-LM). *p *values determining the level of statistical significance are mentioned for all groups. MALAT1-down: KRIB cells with downregulated MALAT1. NS: control cells with non-specific miRNA inhibitors. miR-202: cells with miR-202 inhibitors.

After establishing a possible involvement of lncRNA MALAT1 in lung metastasis of osteosarcoma and finding possible miRNA targets of MALAT1, we turned to in vivo models for further verification. We injected KRIB cells intravenously in mice and counted the number of lung metastatic nodules in control versus cells that had MALAT1 downregulated. The number of metastatic nodules were significantly decreased in the cells with MALAT1 downregulated (Fig. 5A). Moreover, since miR-202 was the most significantly regulated miRNA by MALAT1, we downregulated this miRNA in MALAT1 downregulated KRIB cells. Such decrease in the levels of miR-202 mimicked rescue of MALAT1 and attenuated the effects of MALAT1 downregulation significantly (*p* < 0.0007, Fig. 5A). Since miR-202 is tumor suppressive, as per the results presented so far, we transfected miR-202 in the cells before injecting them in mice and found that higher miR-202 levels by themselves significantly decreased the lung metastatic nodules (Fig. 5A). Finally, as presented above, miR-34a is another miRNA that is regulated by MALAT1 (Fig. 4). We checked if this miRNA could also similarly impact lung metastasis. When we injected cells with ectopic miR-34a, we found a decrease in the lung metastatic nodules but the change was not found to be statistically significant (Fig. 5A).

Having established a role of miR-202 in MALAT1 induced metastasis, we wanted to confirm whether its levels are decreased in patients with lung metastasis. This assumption was based on the inverse relationship between MALAT1 and miR-202 and the elevated levels of MALAT1 in osteosarcoma patients with lung metastasis. We indeed found significantly reduced (*p* < 0.0001) miR-202 levels in patients with lung metastasis relative to patients without metastasis (Fig. 5B). Further, we checked the levels of MALAT1 in osteosarcoma patients versus archived samples from individuals with no osteosarcoma diagnosis. As presented in Fig. 5C, we found elevated MALAT1 levels in osteosarcoma patients and these levels were ~ 7 times higher in the patients. This points to diagnostic value of MALAT1 in osteosarcoma.

In addition to the KRIB i.v. model, we also tested an alternative in vivo model in which we created SAOS-2 that metastasize to lungs. This was accomplished by i.v. injections of SAOS-2 cells successively in mice after collection of lung metastatic nodules. We questioned if such sequential culturing of cells that preferentially metastasize to lungs (SAOS-2-LM) will also show evidence of MALAT1-miR-202 signaling. To test this, we compared parental SAOS-2 cells with SAOS-2-LM cells. When we checked for MALAT1 expression, we found it to be significantly elevated in SAOS-2-LM cells (Fig. 5D). Further, we also found miR-202 to be significantly downregulated in SAOS-2-LM cells (Fig. 5E), as would be expected in cells with elevated MALAT1.

## Discussion

The progress in clinics for the treatment of osteosarcoma patients has been tremendous. A good measure of treatment efficacy is the 5-year survival rate. For osteosarcoma, the 5-year survival used to be ~ 20% until the introduction of adjuvant chemotherapy in 1970s which substantially raised the 5-year survival of osteosarcoma patients to ~ 50%^2,16^. As of now, this 5-year survival has further improved to be ~ 70–80%^8^. Another parameter of progress is the orthopedic involvement. Before the defined role of adjuvant chemotherapy in mid-1970s, orthopedic amputation was often the routine treatment, particularly for high grade osteosarcomas. However, within a time period of over a decade, by around 1990s, the focus of the management of osteosarcoma patients, even those with high grade tumors, shifted to limb salvage^2^.

The focus of our work on lncRNAs is supported by several reports that have documented role of lncRNAs in cancer progression and metastasis^17–21^. In osteosarcoma as well, there are many reports on lncRNAs^19,22–27^. Based on these evidences, it is clear that lncRNAs can influence proliferation and metastasis of osteosarcoma cells. They also seem to have some clinical utility as biomarkers^20,22^, however, no study has so far focused on specific metastasis of osteosarcomas, such as, the lung metastasis of osteosarcoma, the focus of our work. In our study, we started with screening of several potential lncRNAs that could serve as biomarkers for lung metastatic osteosarcomas. To make this exercise worthwhile, we first evaluated patients-derived samples. Our results indicated MALAT1 to be the most dysregulated lncRNA in osteosarcomas with lung metastasis. This was the conclusion based on analysis of all lncRNAs that were either up or downregulated. To establish a mechanistic working model, we next checked for levels of MALAT1 in the osteosarcoma cell lines. Interestingly, we found KRIB cells to have the highest MALAT1 levels among the tested cell lines. This cell line is known to be more metastatic than the other cell lines tested and thus higher levels of MALAT1 in KRIB makes supports our hypothesis and the results from patient samples-based evaluation. Further, among the osteosarcoma cell lines that we tested, U2OS are poorly metastatic and thus lower MALAT1 levels in these cells also make sense. In our study, we also found MALAT1 levels to be higher in osteosarcoma, compared to healthy individuals which means that MALAT1 can not only serve as biomarker for lung metastasis but as a possible diagnostic marker for osteosarcoma.

Identification of an lncRNA signature for lung metastatic osteosarcoma is itself a significant advancement in our knowledge. However, we further studied the mechanism by evaluating the possible role of miRNA sponging by MALAT1. lncRNAs mode of functioning through sponging of miRNAs is well known^23,24,27,28^. In our screening, which involved miRNAs documented to be targets of MALAT1 as well as some revealed by online prediction tools, the two miRNAs that clearly stood out were miR-202 and miR-34a. Among them, the most relevant target of MALAT1, atleast in our model system turned out to be miR-202 as this miRNA was found to be the most significantly regulated by MALAT1. We found MALAT1 to be most significantly upregulated lncRNA in patients with lung metastasis. Since lncRNA sponging of miRNAs means a mutually negative correlation, we expected the sponged miRNAs to be upregulated in our model that comprised of downregulated MALAT1. Thus, the target miRNAs of MALAT1 are expected to be tumor suppressive. A survey of literature supported tumor suppressive role of miR-202^29^, including in osteosarcoma^30^.

Based on the results that are presented, it is quite apparent that MALAT1 and miR-202 unique signature for lung metastatic osteosarcoma. MALAT1 is upregulated in lung metastatic osteosarcomas and thus miR-202 is down-regulated. Moreover, when MALAT1 is downregulated, it leads to much reduced lung metastases of otherwise metastatic osteosarcoma cells. To further evaluate the role of MALAT1-miR-202 axis, we downregulated miR-202 in MALAT1 downregulated osteosarcoma cells while conducting the in vivo studies. The rationale was that if MALAT1 is downregulated, miR-202 should be upregulated and thus its downregulation should attenuate MALAT1 downregulation-mediated effects. Indeed, we found that miR-202 downregulation attenuated effects pf MALAT1 downregulation, thus completely supporting our major conclusions. While our work supports MALAT1-miR-202 as a signature for lung metastasis of osteosarcoma, we can not comment on whether or not this represents a signature unique to lung metastasis. It is possible that MALAT1 might be involved in metastasis of osteosarcomas to other metastatic sites as well. Such evaluations will need to incorporate patient samples that represent metastasis of osteosarcomas to other metastatic sites. Such studies have been initiated in our laboratory and will take some time to mature.

In addition to the in vivo experiment involving highly metastatic osteosarcoma cells that metastasize to lungs, we also tested an alternate model described by Ji et al.^14^. This involves generation of lung metastatic SAOS-2 cells. It is expected that such model would have metastatic signature needed for lung metastasis of osteosarcoma. At the same time, such models are often criticized for not adequately representing clinical disease. Interestingly, we found that compared to parental cells, such generated lung metastatic cells also had significantly higher MALAT1. Also, reciprocally, miR-202 was significantly decreased. These observations further provide corroboration of our major findings.

It needs to be mentioned that while we found MALAT1 to be upregulated in metastatic osteosarcomas and that an oncogenic role of MALAT1 in osteosarcoma is supported by many reports^23,27,31–36^, there are indications of a tumor suppressive role of MALAT1^37^. In this specific report, downregulation of MALAT1 was found to increase lung metastases of breast cancer cells^37^. While the study was performed in a different cancer model, the results are exactly opposite of ours. It looks like that MALAT1 might have different effects in different cancers but still such contrasting observations^38^ make it important to comprehensively evaluate the role of MALAT1 before advancing the findings further in the clinics.

In summary, we provide evidence for a role of MALAT1 in lung metastasis of osteosarcomas which involves regulation of miR-202. It is possible that targeted downregulation of MALAT1 or the upregulation of miR-202 can reduce lung metastasis of osteosarcoma.

## Data Availability

All the data is described within the manuscript.
